# A rare case of intercostal-to-pulmonary artery fistula and its endovascular treatment in the setting of post pulmonary tuberculosis bronchiectasis and haemoptysis

**DOI:** 10.4102/sajr.v29i1.3139

**Published:** 2025-06-20

**Authors:** Lwandile Majozini, Winile Nkosi

**Affiliations:** 1 Department of Radiology, Faculty of Health Sciences, School of Clinical Medicine, University of the Witwatersrand, Johannesburg, South Africa

**Keywords:** intercostal artery-to-pulmonary artery fistula, SA-PAFs, post pulmonary TB bronchiectasis, haemoptysis, digital subtraction angiography, embolisation

## Abstract

Intercostal artery-to-pulmonary artery fistula is an extremely rare variant of systemic artery-to-pulmonary artery fistulas (SA-PAFs). A case of a 38-year-old man presenting with clinically significant haemoptysis secondary to an intercostal artery-to-pulmonary artery fistula in the setting of post-pulmonary tuberculosis (TB) bronchiectasis is described. The fistulae were successfully treated with endovascular coils.

**Contribution:**

This case report illustrates an intercostal artery-to-pulmonary artery fistula associated with post-primary tuberculosis bronchiectasis, highlighting its multimodal radiological features and successful endovascular treatment.

## Introduction

Systemic artery-to-pulmonary artery fistulas (SA-PAFs) are rare arterial malformations characterised by an abnormal communication between a systemic artery and a pulmonary artery.^1^ This anomalous communication can occur between the pulmonary artery and internal mammary, intercostal, bronchial, pericardial or oesophageal arteries.^2^ Intercostal-to-pulmonary artery fistulas are an exceptionally uncommon subtype of SA-PAFs.^3^

The SA-PAFs may be acquired or, in rare cases, occur congenitally. Identified potential acquired causes include inflammatory or infectious processes (e.g. *Mycobacterium tuberculosis* and Actinomycosis), surgical procedures (post-cardiothoracic procedures and post-intercostal chest drain insertion), or neoplastic aetiology.^2^

### Ethical considerations

Ethical clearance for this report was obtained from the University of the Witwatersrand and Human Research Ethics Committee (Medical) (No. R14/49).

## Patient presentation

A 38-year-old man with a history of pulmonary tuberculosis was treated with a 9-month course of anti-tuberculous medication. The patient presented to the medical emergency unit with a 2-week history of non-life-threatening haemoptysis. He had no associated constitutional symptoms or significant medical or family history.

On physical examination, he had stable vital signs with basal crepitations on chest examination. Laboratory investigations revealed worsening anaemia, Hb of 9.8 g/dL, 8.7 g/dL and 6.7 g/dL (13.4 g/dL – 17.5 g/dL) with a normal platelet count of 289 × 10^9^/L, raised inflammatory markers with a C-reactive protein of 230 mg/L, and procalcitonin of 0.26 ug/L. β-d-Glucan (BDG) and Aspergillus galactomannan were negative. Sputum microscopy, culture and sensitivity revealed *Klebsiella pneumoniae*. Sputum GeneXpert test and HIV rapid screen were negative.

**FIGURE 1:**
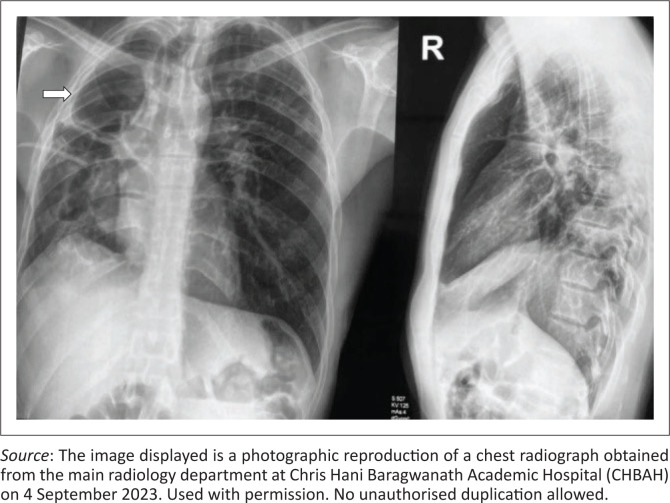
Chest radiographs (frontal and lateral projections) depicting right upper lobe fibrocavitary changes (white arrow) with ipsilateral tracheal deviation.

**FIGURE 2:**
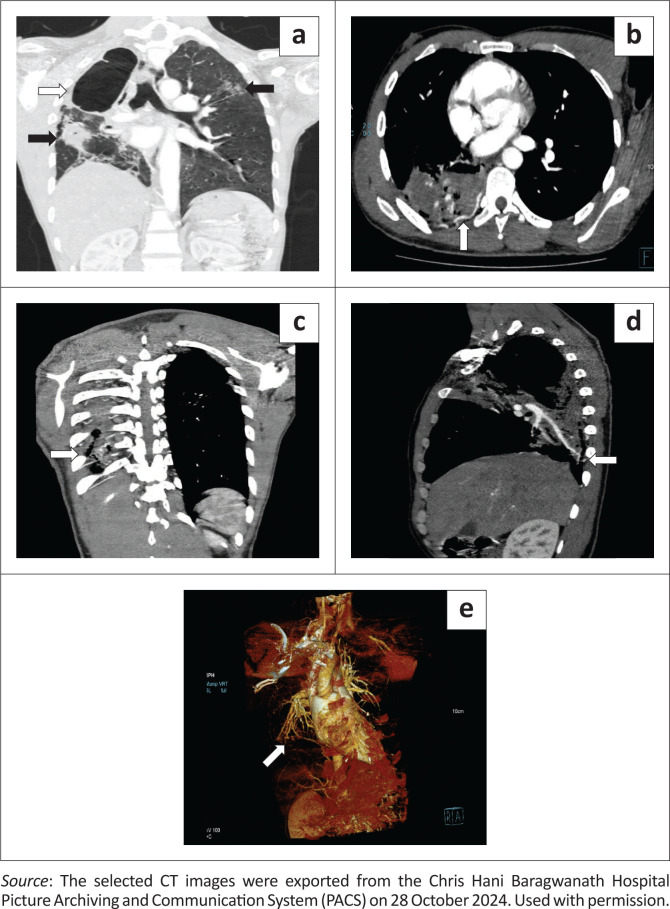
(a) Coronal reconstruction of the contrast-enhanced CT of the chest (lung window) demonstrating the right upper lobe fibrocavitary changes (white arrow) with ipsilateral lung volume loss, tracheal deviation, and opacities in the left upper, and right mid-lung zones with right lower lobe consolidation (black arrows). (b) Axial contrast-enhanced maximum intensity projection (MIP) CT of the chest demonstrating a hypertrophied and tortuous right 9th intercostal artery (white arrow). (c) Coronal contrast-enhanced MIP CT of the chest. (d) Sagittal contrast-enhanced MIP CT of the chest. (e) 3D coronal CT angiogram of the thorax further demonstrates dilatation and tortuosity of the 9th intercostal artery (white arrow).

The chest radiograph revealed right lung upper lobe fibrocavitary changes with bronchiectatic changes and right lung volume loss with ipsilateral tracheal deviation (Figure 1). CT of the chest confirmed the chest radiograph findings of right upper lobe cavitation, fibrotic changes, secondary traction bronchiectasis and associated right lung volume loss with patchy ground glass opacities in the right mid and bilateral upper lung zones. No mycetoma or intracavitary air-fluid level were demonstrated (Figure 2a). In addition, the chest CT demonstrated hypertrophied and tortuous right bronchial arteries and right lower intercostal arteries with no Rasmussen’s aneurysm (Figure 2b–e).

**FIGURE 3:**
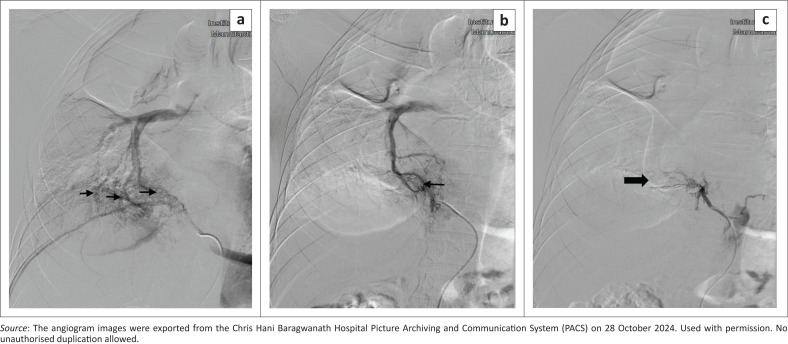
Selective digital subtraction angiogram of the right 9th intercostal artery showing a fistulous connection between the right 9th intercostal artery and right lower lobe pulmonary artery tertiary branches (black arrows), (a) pre-embolisation, (b) during embolisation and (c) completion arteriogram demonstrates no remaining communication of the intercostal artery-to-pulmonary artery fistula after coil embolisation (black arrow).

Due to ongoing haemoptysis and worsening anaemia, the patient was then referred to the interventional radiology team for possible bronchial artery embolisation (BAE). Digital subtraction angiography revealed multilevel abnormal fistulous connections between the 7th and 9th intercostal arteries and the right lower lobe pulmonary arterial tertiary branches. A fistulous connection was demonstrated between the right 9th intercostal artery and the tertiary branches of the right lower lobe pulmonary artery (Figure 3a–b). Subsequently, transcatheter coil embolisation of the right 7th–9th intercostal arteries, as well as bronchial arteries (not shown), was successfully performed with favourable angiographic results (Figure 3c).

While admitted to the medical ward, the patient received two units of blood and a 5-day course of meropenem. The patient recovered and was subsequently discharged, to be followed up in the Respiratory outpatient clinic.

## Discussion

Intercostal-to-pulmonary arterial fistulas are extremely rare, with very few cases documented in the literature.^3^ To the best of the authors’ knowledge, this is the first case report from sub-Saharan Africa that showcases the use of multimodal imaging to identify an intercostal artery-to-pulmonary artery fistula and its endovascular treatment in the context of post-primary tuberculosis (TB) bronchiectasis.

Patients with SA-PAF may not exhibit any symptoms and can thus be identified incidentally.^4^ Symptomatic patients may present with complications such as bacterial infection, haemorrhage from rupture, pulmonary arterial hypertension and congestive cardiac failure.^5^ Given the substantial risks of cardiovascular consequences such as endocarditis, pulmonary arterial hypertension and congestive heart failure, it is critical to identify and address these fistulae.^5,6^

Chest radiography and clinical evaluations can effectively identify post-TB sequelae; however, they frequently overlook the critical presence of vascular lesions.^7^ In the context of an intercostal-to-pulmonary artery fistula, a chest radiograph may show pulmonary infiltrates, increased vascular markings, pleural thickening, and rib notching.^2^

Reported CT imaging findings include pleural thickening and nodular or diffuse soft tissue opacity in the subpleural lung abutting the pleura.^8,9^ Unlike for bronchial arteries, there is no standard size cut-off for intercostal arteries. Therefore, asymmetric enlargement of the intercostal arteries may help identify hypertrophy.^10^

Ventilation-perfusion (V/Q) scans and pulmonary artery blood gas measurements can be used as ancillary tests to evaluate the haemodynamic effects of a SA-PAF. Systemic-to-pulmonary artery fistulas initially create a left-to-right shunt, which increases pulmonary blood flow and pressure. Over time, this can result in elevated pulmonary vascular resistance, potentially reversing the direction of the shunt to right-to-left.^11^ In a right-to-left shunt, blood bypasses the lungs, leading to a V/Q mismatch. This leads to a decrease in PaO_2_, an increase in the alveolar-arterial (A–a) gradient, and elevated levels of PaCO_2_.^12^

The definitive diagnosis is established through selective digital subtraction angiography; however, CT angiography with three-dimensional reconstruction is valuable for identifying abnormal vessels.^13^ In cases of life-threatening haemoptysis, combining CT with fibreoptic bronchoscopy enhances diagnostic accuracy and facilitates targeted therapeutic interventions. The importance of fibreoptic bronchoscopy is highlighted in maintaining airway control, particularly for patients with bilateral lung disease.^14^

Haemoptysis in the context of previously treated tuberculosis can arise from various causes, including bronchiectasis, reactivation of tuberculosis, scar carcinoma, aspergillomas or mycetomas, broncholiths, pulmonary cavities, and vascular complications such as pseudoaneurysms (e.g. Rasmussen’s aneurysm).^15^

At present, guidelines or recommendations for managing patients with post pulmonary TB haemoptysis are sparse.^7^ Treatment often relies on hospital protocols derived from international publications, which may not address the unique needs of the patient population. Implementing tailored local guidelines is essential for ensuring optimal care and improving patient outcomes. Embolisation is currently utilised as a less invasive treatment option, while surgical intervention remains a crucial treatment alternative.^5^

The patient in this case report experienced worsening and persistent haemoptysis, which necessitated a blood transfusion. Chest radiographs and CT chest imaging did not initially clarify the patient’s presentation and given the persistent haemoptysis, selective intercostal artery angiography was crucial in identifying the source of the bleeding. However, in retrospect, there was a suspicion of an intercostal artery-to-pulmonary artery fistula based on the CT chest imaging findings. The angiogram revealed multilevel intercostal arteries-to-pulmonary artery fistula and hypertrophied bronchial arteries which were subsequently embolised via a transarterial approach using detachable coils for the fistulas and polyvinyl alcohol (PVA) particles for the hypertrophied bronchial arteries, leading to a positive and favourable clinical outcome.

## Conclusion

Intercostal artery-to-pulmonary artery fistula is a rare but significant vascular malformation that must be recognised as a potential hidden cause of ongoing haemoptysis, especially in patients with post-primary tuberculosis. Early recognition and initiating appropriate treatment including endovascular treatment can dramatically improve patient outcomes and prevent life-threatening complications.
